# When the Music Comes From the Brain: A Rare Case of Auditory Seizures Secondary to a Right Temporal Lobe Arteriovenous Malformation

**DOI:** 10.7759/cureus.57932

**Published:** 2024-04-09

**Authors:** Wilson Rodriguez, Navreet Chennu, Lissette Orozco, Gunjanpreet Kaur, Jafar Kafaie

**Affiliations:** 1 Neurology, Saint Louis University School of Medicine, Saint Louis, USA; 2 Internal Medicine, Trinity Health Ann Arbor Hospital, Ypsilanti, USA; 3 Neurology, Saint Louis University Hospital, Saint Louis, USA

**Keywords:** temporal lobe, stereotactic radiosurgery, musical hallucinations, avm, auditory seizures

## Abstract

Focal seizures with subjective auditory phenomena, known as auditory seizures, are uncommon and can include simple to complex auditory hallucinations. We present a case of a 59-year-old man who presented with motor and non-motor seizures. He had a four-month history of hearing things resembling continuous metallic sounds, pennies dropping into a bank, persistent music after radio cessation, and the sound of a passing train. Brain MRI showed multiple serpiginous flow voids in the right temporal lobes, consistent with an arteriovenous malformation that was confirmed eventually with a diagnostic brain angiogram. The etiology of the seizures was related to a structural lesion in the setting of a right temporal arteriovenous malformation (AVM). Treatment with 2000mg of levetiracetam twice daily and 300mg of oxcarbazepine twice daily improved symptoms, and subsequent stereotactic radiosurgery ablation successfully treated the AVM. Post-treatment MRI showed reduced visibility of parasitized vessels, with controlled generalized seizures but partial control of auditory seizures.

## Introduction

Arteriovenous malformations (AVMs) are abnormal connections between arterial and venous blood vessels that can occur in various locations within the brain. These malformations disrupt normal cerebral blood flow, leading to a range of symptoms, with seizures being a common manifestation. The semiology of seizures can vary depending on the location within the brain. While many patients with AVMs experience tonic-clonic seizures (56.7%), others may present with simple partial (20%), complex partial (3.3%), or partial seizures with secondary generalization (20%) [1]. Focal seizures characterized by subjective auditory phenomena (AP), often referred to as "auditory seizures (AS)", are relatively rare, affecting approximately 2-7% of individuals with epilepsy. AP can manifest as simple noises, complex auditory hallucinations, or sound distortions during ictal periods [2]. Musical hallucinations, categorized into aura/ictal, post-ictal, and inter-ictal phenomena, remain poorly understood. While AS are traditionally associated with temporal lobe origins, their lateralization remains unclear [3].

We report a rare case involving a patient diagnosed with an AVM in the right temporal lobe, manifesting generalized motor seizures alongside focal AS marked by auditory hallucinations. This case underscores the significance of recognizing auditory hallucinations as a symptom attributable to temporal lobe lesions, emphasizing the need for comprehensive assessment and management strategies.

## Case presentation

A 59-year-old man with a medical history of diabetes mellitus type 2, hypertension, and atrial fibrillation was admitted to the emergency department due to transient episodes of altered mental status and abnormal movements suggestive of seizures.

Initially, the patient experienced episodes characterized by staring and clenching of his left extremity to his chest, accompanied by an inability to follow commands. Post-ictally, he exhibited drowsiness and had no recollection of the events. The episodes began approximately four months before the patient's presentation, typically lasting 2 to 3 minutes and occurring two to three times per month. Additionally, he reported auditory hallucinations resembling continuous metallic sounds, pennies dropping into a bank, persistent music after radio cessation, and the sound of a passing train. He reported experiencing these episodes three times a week, with durations varying from mere seconds to up to two minutes. Visual hallucinations of snakes and bugs were also reported by the patient, but it was just one episode and happened before coming to our institution.

**Figure 1:**
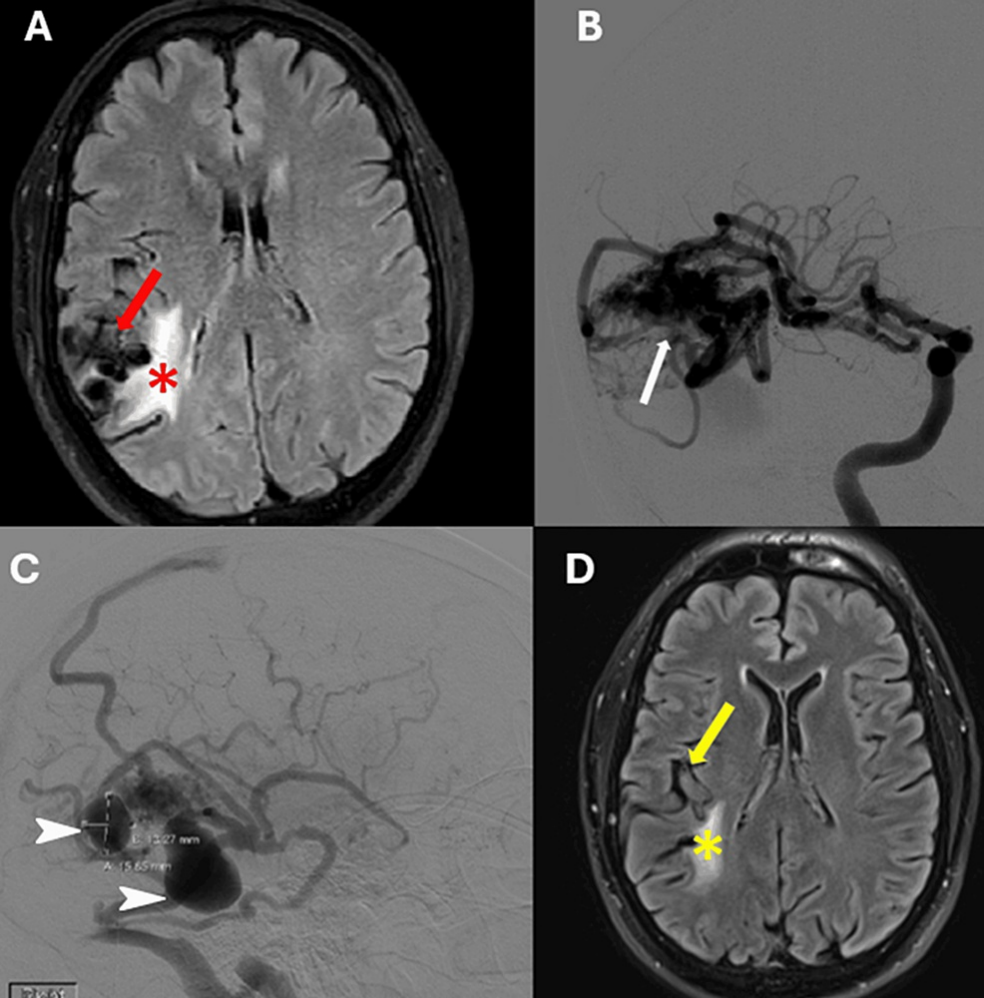
Brain MRI and Diagnostic Angiogram A: Axial FLAIR sequence of the brain showing multiple serpiginous flow-voids in the right perisylvian temporal lobe (red arrow) consistent with an arteriovenous malformation, and perilesional hyperintensity suggestive of gliosis from steal phenomena (red asterisk). B-C: DSA showing an arteriovenous malformation (B, white arrows) with main feeder from a trifurcation branch of the right MCA, and two giant varices suggestive of venous pseudoaneurysm (C, white arrowheads). D: Axial FLAIR sequence of brain MRI after one year showing right temporal lobe AVM with decreased conspicuity of parasitized vessels (yellow arrow) and decreased perilesional gliosis (yellow asterisk). FLAIR: Fluid-attenuated inversion recovery; DSA: digital subtraction angiography

Initial computed tomography of the head (CT) revealed a right temporal lobulated hypodensity lesion. Subsequent brain MRI with and without contrast demonstrated multiple serpiginous flow voids in the right perisylvian parietal and temporal lobes, consistent with an AVM (Figure 1A). Continuous EEG monitoring revealed right temporal epileptiform discharges and right temporal slowing. Diagnostic brain angiography confirmed a Spetzler-Martin grade 3 right temporal AVM with feeders originating from the right middle cerebral artery (MCA) (Figure 1B) and two venous pseudoaneurysms (Figure 1C). Treatment commenced with intravenous 1000 mg of levetiracetam twice daily, later increased to 2000 mg twice daily. Despite persistent symptoms, oral oxcarbazepine 300mg twice daily was added, leading to further control of motor seizures and decreased frequency of AS. Two months post-diagnosis, the patient underwent stereotactic radiosurgery (SRS) ablation with a total dose of 2400 cGy administered in three fractions to eradicate the AVM, without any complications. Subsequent brain MRI performed five months post-SRS revealed decreased conspicuity of parasitized vessels (Figure 1D).

Clinically, his motor seizures responded well to medical therapy and SRS; however, his AS were partially controlled occurring three to four times per year. The patient continues taking oral oxcarbazepine 300mg twice daily and levetiracetam 2000mg twice a day. 

## Discussion

Temporal lobe AVMs are characterized by abnormal connections between arterial and venous vasculature, leading to a variety of symptoms determined by their localization. Subtypes of temporal lobe AVMs include lateral temporal lobe, medial, sylvian, and ventricular, ranked in decreasing order of occurrence [4]. In this case, the patient's AVM was classified as Spetzler-Martin grade 3, with feeders originating from the right MCA.

The patient experienced seizures of two distinct semiologies. The first semiology manifested as focal motor seizures with impaired awareness and secondary generalization. The second semiology presented as focal non-motor auditory seizures with auditory hallucinations. Auditory hallucinations were described as a continuous metallic sound resembling a penny dropping into a bank, the passing sound of a train with varying speeds, and metallic clicking noises. Additionally, the patient reported experiencing an alteration in the tempo and volume of music he was listening to, which appeared faster and louder before returning to normal. Visual hallucinations of snakes and bugs were reported initially but had resolved by the time the patient presented to our institution. The patient underwent continuous EEG monitoring, which revealed epileptiform discharges and focal slowing originating from the right temporal lobe during his auditory phenomena. This finding aligns with existing literature, as auditory symptoms often lack a clear ictal EEG correlation. Instead, diagnosis is typically based on interictal correlation and response to antiseizure medication [3]. 

The cerebral steal phenomenon emerges as a crucial mechanism to consider in the development of the second semiology involving visual and auditory hallucinations. An MRI performed on this patient revealed a high signal intensity on T2 near the right insula, situated at the junction of the temporal and parietal lobes, indicative of potential gliosis that could have been caused by ischemia. A parallel case report documenting auditory hallucinations posited that the modified function of the medial temporal and mesolimbic pathway could arise from metabolic alterations resulting from shifts in blood flow or structural reorganization [4,5].

The patient initially received an intravenous regimen of 1000 mg of Levetiracetam twice daily for seizure control before being transferred to our institution. Despite this, he persisted with musical hallucinations, prompting an escalation to 1500 mg twice daily, and subsequently to 2000 mg twice daily. This adjustment successfully alleviated the auditory hallucinations and reduced the intensity of mechanical sounds. To further enhance seizure management, oral oxcarbazepine at a dosage of 150 mg twice daily was introduced. Upon discharge, the patient's medication regimen comprised oxcarbazepine at 150 mg twice daily and levetiracetam at 2000 mg twice daily. Despite these measures, the patient experienced residual auditory hallucinations post-discharge, leading to an increase in oxcarbazepine dosage to 300 mg twice daily. Additionally, the patient underwent SRS ablation with a total dose of 2400 cGY administered in three fractions. It appears that focal seizures featuring auditory phenomena pose challenges in terms of treatment, as evidenced by previous case reports. Similarly, in our patient, achieving control over these seizures required further titration of two antiseizure medications [3].

The standard treatment approach for AVMs relies on the Spetzler-Martin grading system, with lesions graded IIIB, IV, and V typically managed through hypofractionated SRS, while lesions with lower grades are addressed using single-fraction SRS [6]. The primary objective of SRS therapy for smaller AVMs is to mitigate the risk of hemorrhage and prevent the progression of associated complications, including seizures [7]. Seizures represent the second most common presentation of AVMs after hemorrhage, particularly among cases involving unruptured malformations.

A study conducted by Ding et al., involving 229 patients who underwent radiosurgery for temporal lobe AVMs with an initial seizure presentation, revealed notable findings. Among these patients, 132 (57.6%) experienced improvements in seizure control, with 46 (20.1%) achieving complete seizure remission and 86 (37.6%) experiencing a reduction in seizure frequency. Remarkably, only 4% of patients experienced worsening seizures, suggesting that prophylactic antiepileptic regimens may not be universally necessary [8].

Our patient's case serves as a pertinent example wherein effective seizure management was achieved through the administration of oxcarbazepine and levetiracetam following SRS ablation.

## Conclusions

In this case report, our goal is to underscore the significance of considering temporal lobe lesions as a potential etiology for focal seizures accompanied by auditory phenomena such as musical hallucinations. While electrographic studies play a crucial role, the absence of clear ictal correlations should not deter clinicians from promptly diagnosing and initiating early treatment with antiseizure medication. Further research is imperative to deepen our understanding of the pathophysiology underlying auditory seizures and the challenges associated with their management using antiseizure medications.
